# Evaluation of Palatal Furcation Groove and Root Canal Anatomy of Maxillary First Premolar: A CBCT and Micro-CT Study

**DOI:** 10.1155/2021/8862956

**Published:** 2021-01-08

**Authors:** Xiaojing Liu, Meili Gao, Qingxia Bai, Jianping Ruan, Qun Lu

**Affiliations:** ^1^Clinical Research Center of Shaanxi Province for Dental and Maxillofacial Diseases, Department of Preventive Dentistry, College of Stomatology, Xi'an Jiaotong University, XiWu Road 98, Xi'an, 710004 Shaanxi Province, China; ^2^State Key Laboratory of Military Stomatology & National Clinical Research Center for Oral Diseases & Shaanxi Key Laboratory of Stomatology, Department of Operative Dentistry and Endodontics, School of Stomatology, Air Force Medical University, West Changle Road 145, Xi'an 710032, China; ^3^Department of Stomatology, Yulin First Hospital, Yuxi Avenue 93, Yulin, 719000 Shaanxi Province, China; ^4^Department of Biological Science and Engineering, The Key Laboratory of Biomedical Information Engineering of Ministry of Education, School of Life Science and Technology, Xi'an Jiaotong University, Xi'an 710049, China

## Abstract

**Objectives:**

This study is aimed at investigating the root and root canal morphology by cone beam computed tomography (CBCT) and palatal furcation groove of the buccal root by microcomputed tomography (micro-CT) of maxillary first premolars in a Chinese subpopulation.

**Methods:**

This study assessed CBCT images of 440 patients aged 14-80 years. Based on Vertucci's classification, the number of roots and the canal configuration were determined. Forty-eight maxillary first premolars with furcation grooves were analyzed by micro-CT in patients aged 18-25 years.

**Results:**

Based on the CBCT assay, 70.22% and 29.32% of maxillary first premolars were 1 root and 2 roots, respectively. The configuration indicated statistical difference (*P* < 0.05) between male and female patients. The most common canal type was type IV and was found in 44.32% of cases, followed by type I in 27.84%, and then type II in 20.57%. Root bifurcations had 40.13% prevalence which was distributed more in the middle third than in the cervical and the apical third. For the micro-CT study, 95.83% of the furcation groove configuration was found in the bifurcated maxillary first premolars. The length varied from 1.02 to 7.63 mm. The mean depth of this groove was 0.57 mm in the root coronal, 0.47 mm in the root middle, and 0.22 mm in the root apical level. Palatal dentin width was smaller than 1 mm.

**Conclusion:**

The anatomy of the root and root canal system and the irregular wall width of maxillary first premolars with furcation grooves may help dentists to understand the anatomical morphology and improve the outcomes of endodontic treatment.

## 1. Introduction

Teeth play a crucial role in performing the mastication function. Tooth morphology is an essential part of stomatology. It is vital to understand and to master the appearance and the internal structure of human teeth in various fields for stomatology. Recognition of the possible changes in the root and root canal anatomy of teeth may reduce the possibility of overlooking the root canal system during treatment [1]. Among teeth, maxillary first premolars exhibit a complicated root anatomy, such as bifurcated roots, narrow furcation entrance, deep mesial concavities, and complicated canal configurations according to Vertucci's classification. These morphological changes may affect the results of root canal therapy and periapical surgery. Lack of understanding of teeth morphological variability and complex anatomy may lead to procedural accidents [2–4].

The root and root canal morphology of the maxillary first premolar is complex. When looking for the root canal orifice, it is easy to have root omission. Besides, incomplete root canal filling and broken instruments may occur easily during preparation and obturation. The maxillary premolars are common candidates for root canal treatment and account for 15.8%-21.5% of all treated teeth [5]. In our previous paper, we analyzed the frequency of root canal morphology in the adolescent population [6]. However, the morphological variation of the root canal system is presented due to the differences in geologic region, ethnicity, and age. The huge information on root canal frequency warrants further investigation in a broad range of age of people.

Considering the features of root canal morphology, furcation grooves were often observed in the buccal root of 2-rooted maxillary first premolars. Currently, few studies mention the presence of this groove or invagination on the palatal aspect of the buccal root. This groove was previously described as a “developmental depression,” “buccal furcation groove,” or “furcal concavity.” Reports have indicated that 62%~100% of the buccal roots in the maxillary first premolars had this structure [2, 3, 7]. During the root canal treatment and postcanal preparation, root canal perforation and crack would appear with high possibility because of the weak root canal wall in this groove, which affects the prognosis of teeth [8–10]. It is easy to accumulate plaque in this groove, but difficult to reach with curettage equipment. Once suffering from periodontal disease, the prognosis is poor. So, this groove has been described as a “danger zone” [11].

Therefore, the objective of this study was to investigate the anatomy of the root and root canal system using cone beam computed tomography (CBCT) technique in a broader age population. Additionally, an anatomic study of furcation grooves was analyzed by using high-resolution computed tomography (micro-CT) to obtain a higher spatial feature in a Chinese adolescent population.

## 2. Materials and Methods

### 2.1. Samples

For CBCT investigation, 880 CBCT images of maxillary first premolars (440 bilateral) were selected from a Chinese subpopulation of 440 patients (212 men and 228 women) aged 14-80 years for dental diagnosis or treatment planning at Yulin First Hospital in Yulin City, Shaanxi Province, China. For palatal furcation grooves using the micro-CT technique, 48 extracted maxillary first premolars from the adolescent population (aged 15-25 years) were used, and they were cleaned in 3% hydrogen peroxide. Teeth selected in this study correspond with the following criteria: (1) complete teeth without fracture, (2) mature teeth with fully developed root, and (3) teeth without root canal fillings, posts, or restoration. This investigation was performed according to the Declaration of Helsinki and approved by the Ethics Committee of Yulin First Hospital.

### 2.2. CBCT Scanning and Analysis

CBCT images were obtained by Kavoi-CAT CBCT (KaVo, USA). The CBCT unit was operated at 120 kV and 5 mA with an exposure time of 27 seconds, resolution of 0.125 mm voxels, and field of view (13 cm height and 16 cm diameter). CBCT images were analyzed by iCAT Vision software (Imaging Sciences International Co. Ltd., USA). The patient's name, gender, age, and tooth position were recorded.

**Figure 1:**
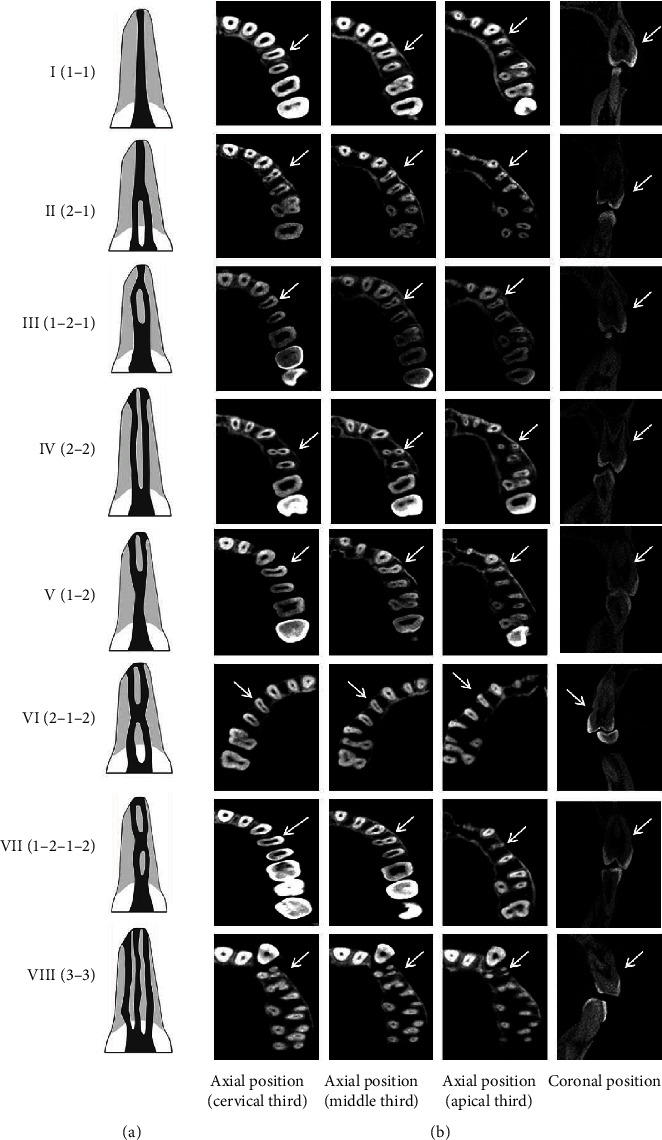
Classification of root canal system according to Vertucci's classification (a) and CBCT images showing root canal configuration of maxillary first premolars (b). Arrows indicate the examined teeth.

All teeth were evaluated in axial, coronal, and sagittal planes. The number of roots was recorded in each tooth. The location on the coronal, middle, and apical third of root bifurcations was recorded in the maxillary first premolars. For root canal analysis, according to Vertucci's root canal classification, root canal configurations were classified into eight types: type I (1-1), type II (2-1), type III (1-2-1), type IV (2-2), type V (1-2), type VI (2-1-2), type VII (1-2-1-2), and type VIII (3-3) (Figure 1(a)) [1].

### 2.3. Micro-CT Measurement

The teeth which had a buccal root in 2-rooted maxillary first premolars were scanned by a micro-CT scanner (Siemens Inveon MM Gantry CT, Germany) with an isotropic resolution of 14.97 *μ*m and exposure time of 500 ms at 80 kV and 500 *μ*A. Next, the Mimics 10.01 software (Materialise, Leuven, Belgium) was used for the 3-dimensional analysis.

**Figure 2:**
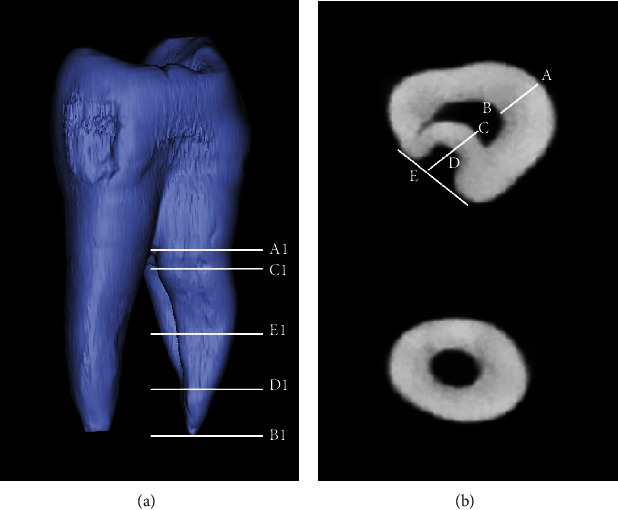
Measurement of the buccal root with furcation grooves in maxillary first premolars using micro-CT. (a) Vertical measurement. (b) Horizontal measurement. A1: root furcation position; B1: apical position; C1: the start point of furcation groove; D1: the end point of furcation groove; E1: the middle point of CD connection. A1B1: buccal root length; C1D1: furcation groove length; A1C1: length from root bifurcation to the beginning of furcation groove; B1D1: length from apical position to the end point of furcation groove. AB: buccal root canal wall thickness; CD: palatal root canal wall thickness; DE: furcation groove depth (depth of root invagination).

From the root tip along the long axis of the buccal root to the root bifurcations, the morphological changes of the anatomical palatal groove were measured in the vertical and horizontal directions. Setting of the reference points (Figure 2(a)) was as follows: A1: root furcation position; B1: apical position; C1: the start point of furcation groove; D1: the end point of furcation groove; and E1: the middle point of C1D1 connection. The data, including lengths of A1B1, C1D1, A1C1, and B1D1, were measured. In Figure 2(b), the widths (thickness) of AB and CD and the furcation groove depth of DE were determined in the buccal root with furcation groove. Each parameter is measured three times, and the average value is taken.

### 2.4. Statistical Analysis

SPSS 17.0 software was used for statistical analysis. The chi-square test was used to compare the root types of the first premolar in different genders and positions. The paired *t*-test was used to compare the thickness of the root canal in different directions of the same horizontal section. *P* values < 0.05 were considered statistically significant.

## 3. Results

### 3.1. Frequency of the Different Roots by CBCT in the General Population

**Figure 3:**
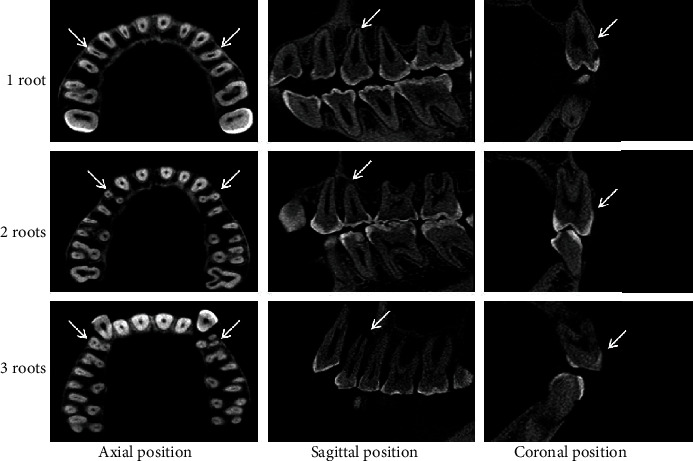
CBCT images showing root types of maxillary first premolars. Arrows indicate the examined teeth.

The frequency distribution of the root number was indicated as 1 root, 2 roots, and 3 roots (Figure 3) in maxillary first premolars, with the corresponding prevalence of 70.22%, 29.32%, and 0.46%, respectively (Table 1). There was a significant difference in the prevalence of 1-rooted and 2-rooted teeth between men and women (*P* < 0.05). On the other hand, no significant differences were observed in the prevalence of 1 root, 2 roots, and 3 roots between the left and right sides (*P* > 0.05) (Table 1).

### 3.2. Frequency of the Root Bifurcation and Furcation Groove in Buccal Root by CBCT in the General Population

**Figure 4:**
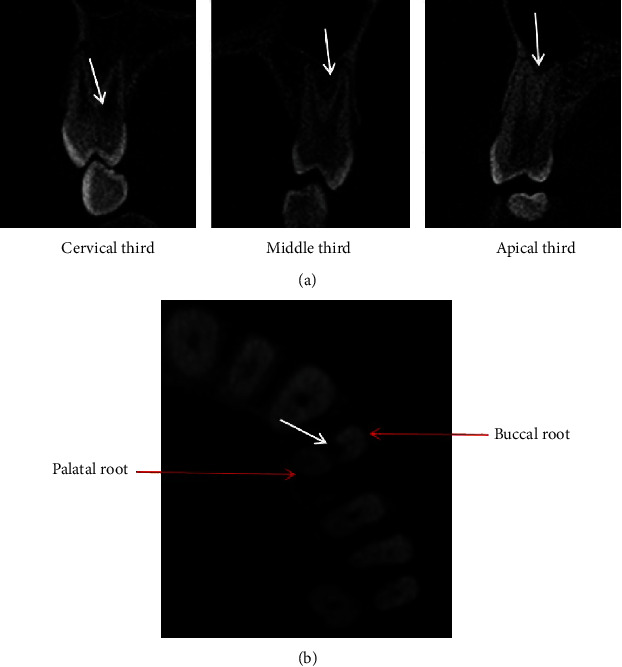
CBCT images showing the level of root bifurcations (a) and furcation groove (white arrow, (b)) in maxillary first premolar. White arrows (a) indicate the positions of coronal third, middle third, and apical third.

Root bifurcations were also found in the analyzed maxillary first premolars (Figure 4(a)). The prevalence of root bifurcations was 40.11% (353/880) in maxillary first premolars. According to the position of bifurcations, they were divided into the cervical, middle, and apical third (Figure 1) levels. The prevalence of bifurcation in the three levels was 9.92% (35/353), 62.32% (220/353), and 27.76% (98/353), respectively. In the analyzed teeth, the palatal furcation groove existed in the buccal root of 2-rooted maxillary first premolars (Figure 4(b)).

### 3.3. Root Canal Types by CBCT in the General Population

Based on the Vertucci classification (Figure 1(a)) [1], there were eight root canal types (Figure 1(b)) in the analyzed 880 maxillary first premolars. The prevalence of 1-rooted canal (type I) was 27.84%, that of the 2-rooted canal (types II-VII) was 71.70%, and that of the 3-rooted canal (type VIII) was 0.46% (Table 2). Moreover, as shown in Figure 4 and Table 2, in the 1-rooted maxillary first premolars, the most common canal configuration was type I (27.84%), followed by type II (20.57%), and then type IV (15.23%). The canal configuration was type IV (29.09%) in 2-rooted maxillary first premolars. Simultaneously, the most common canal configuration was type IV (44.32%) in maxillary first premolars (Table 2; Figure 1(b)).

### 3.4. Furcation Groove with Buccal Root by Micro-CT in the Adolescent Population

Because of the palatal furcation groove existing in the buccal roots, we used micro-CT to analyze this structure. In this paper, the vertical (Figure 2(a)) and horizontal lengths (Figure 2(b)) in the furcation groove were additionally determined in the adolescent population (Figure 2). In the 48 extracted maxillary first premolars, 46 teeth had furcation grooves in the palatal aspect of buccal roots and showed a prevalence of 95.83%. Lengths of the furcation grooves ranged from 1.02 to 7.63 mm, with an average of 3.51 mm. The mean groove depths were 0.57 mm in the coronal level, 0.47 mm in the middle level, and 0.22 mm in the apical level. The maximum depths were 1.59 mm, 0.86 mm, and 0.35 mm in the coronal level, middle level, and apical level, respectively. The deepest part of the lateral groove of the palate is located in the direction of the root coronal. The furcation groove depth (DE) gradually became shallower and disappeared at the root apex (Table 3). The depths gradually decrease from the root cervical to the root apical.

The thickness of the buccal (AB) and palatal (CD) canal walls decreases from the root coronal to the root apical. It is worth noting that the thickness of the palatal root canal wall is less than 1 mm in the three levels (Table 4). The thickness of the buccal root canal was significantly greater than that of the palatal root canal (*P* < 0.05).

## 4. Discussion

Nowadays, two main CT techniques are usually adopted and are considered adequate for anatomical analysis in root morphology, that is, CBCT and micro-CT. CBCT is a noninvasive technology which provides an ability to evaluate root canal morphology and produces high-quality 3D diagnostic images without structure overlap [12, 13]. As for the micro-CT, the main advantage that makes it turn into a particularly attractive technique for evaluating root morphology is its higher spatial resolution than that achieved by CBCT [14]. Thus, in the present study, CBCT was used to evaluate root and root canal morphology in the general population. The micro-CT technique was used to assess the anatomic data of furcation grooves in the adolescent samples.

The anatomical morphology of maxillary premolars shows a variety of numbers and types in roots. Ahmad and Alenezi reviewed the various studies, which included a total of 6878 maxillary first premolars [5]. The findings have indicated that most maxillary first premolars show one (41.7%) or two (56.6%) roots. However, our study found that the occurrences of 1 root and 2 roots in maxillary first premolars were 70.22% and 29.32%, respectively. The difference may be due to the higher occurrence of 2-rooted maxillary first premolars in populations in countries other than China, such as the USA, Australia, Brazil, Turkey, Poland, India, Uganda, Saudi Arabia, and Kosovo. As for the occurrence of maxillary first premolars in Chinese populations, Cheng and Yulai [15] found 57.4% of 1 root and 41.5% of 2 roots. Tian et al. [16] also found that the occurrences were seen 66% in 1 root and 33.3% in 2 roots. We may elucidate that in the Chinese population, the occurrence was relatively higher in 1-rooted than in 2-rooted maxillary first premolars. Additionally, a significant gender difference was found in 880 teeth for the percentage of 1 root (male 61.32%, female 78.51%) and 2 roots (male 38.21%, female 21.05%). In another investigation of 442 maxillary first premolars, the similar distributed trend appeared in one root (male 33.58%, female 62.68%) and in two roots (male 62.68%, female 33.33%) [15]. The frequency distribution indicated no statistical significance between the left and right lateral teeth as previously reported in another Chinese population [16]. In the present study, the occurrence of maxillary first premolars with 3 roots is 0.46%, and this is in line with previously reported incidence ranging from 0.4% to 9.2% [15, 17, 18]. Our findings demonstrated that ethnicity and gender are major controlling factors in root development in the Chinese population.

The present study found that the root bifurcations occurred in the cervical third, middle third, and apical third. The findings demonstrated that the roots in maxillary premolars could divide in any third of the root with a greater incidence in the middle third [19]. They may influence prosthesis support, orthodontics, and insertion of dental implants [20]. To avoid the pulp cavity perforation, understanding the level of root bifurcation is essential to obtain the most desirable result during endodontic procedures.

Concerning the internal root canal configuration, the majority of maxillary first premolars had canal configuration type IV (2-2) (73.2%) and had 15.6% configuration type II (2-1) in the Egyptian subpopulation [21]. Similarly, type IV (82.2%), followed by type II (7.7%), and then type I (6.5%) were found in the Brazilian population [19]. Canal configurations of type IV (69.1%), then type I (10.8%), and type II (8.4%) were found in maxillary first premolars of the Saudi Arabian population [22]. In the present study, the most common canal configuration of maxillary first premolars was type IV (44.32%). Then, 27.84% had configuration type I, and 20.57% had configuration type II. In our previous paper, the distributions of type IV, type I, and type II were 52.78%, 25%, and 8.33%, respectively [6]. These studies and our observations suggest that type I and type II showed different configurations in order changes. In contrast, type IV was the most general observation in maxillary first premolars as previously reviewed by Ahmad and Alenezi [5]. Additionally, in the examined maxillary first premolars, configuration of two roots with two canals was 29.09%, which is relatively lower than that in another Chinese population which included 1056 CBCT images from 601 patients [9]. This diversity may be due to the population studied from the northern and southern areas in China. Also, it is relatively lower than that from Brazilian (41.7%) and Indian (46%) populations [18, 23]. In the present study, canal configurations of type I (27.84%) and type II (20.57%) were found in one root canal of the measured maxillary first premolars. However, type II (42.23%) and type I (35.6%) were observed in one-rooted canal teeth [19] in a Brazilian population study which analyzed 496 maxillary first premolars. These findings suggest that the geologic region and ethnicity may affect the frequency distribution for root canal types.

It is interesting to note the external anatomic features, such as root curvature and furcation groove. Two-rooted maxillary premolars exhibit a high prevalence of vertical root fracture because of their bifurcation, cervical constriction, concavities, and furcation grooves in the roots [24]. Thus, furcation grooves should be taken into consideration during dental procedures involving maxillary premolars [5]. Marca et al. found that micro-CT provided images with a spatial resolution higher than that obtained in CBCT [14]. Up to this point, few studies mention the presence of furcation grooves in the palatal aspects of the buccal roots in upper first premolars. In the present study, the furcation groove in the palatal aspect of the buccal root was analyzed by micro-CT technique. The palatal furcation groove was found in 2-rooted maxillary first premolars. The prevalence of the furcation groove was 95.83% in the examined teeth of the adolescent population, which is consistent with the prevalence ranged from 62% to 100% as in previous reports [2, 3, 7]. Our results were similar to the report that this groove starts from apical to the bifurcation and then becomes gradually shallower and disappears toward the apex [2]. In the present study, the average depth mainly fluctuated between 0.22 and 0.57 mm, with a maximum value of 1.59 mm in the coronal level. The findings of depth are larger than those in the study with subjects aged 35 to 65 years [10]. In our paper, the mean distance of 3.51 mm was observed, while a longer mean distance than 5.38 mm was previously reported [2]. Reports have shown that the length of these grooves varied from 1.1 to 9.0 mm, which are relatively shorter than those in our examined teeth, ranged from 1.02 mm to 7.63 mm [5]. In a study of adult subjects aged 35 to 65 years, average widths of 1.17 mm in the coronal level, 0.97 mm in the middle level, and 0.85 mm in the apical level were reported in the palatal canal wall. The average widths of 1.41 mm, 1.13 mm, and 0.77 mm in the three corresponding levels of the buccal canal wall were reported [3]. We observed the means of 0.88 mm, 0.83 mm, and 0.65 mm in the palatal canal wall and 1.33 mm, 1.15 mm, and 0.93 mm in the buccal canal wall, at coronal, middle, and apical levels, respectively. The discrepancies may be due to the different populations and ages in the previous study [3] and in our study. To date, no clinical study has investigated the lengths, widths, and depths of furcation grooves in the palatal aspect of the buccal roots of upper first premolars in North China. The results of this study provide the additional evidence for these anatomic parameters. Considering the canal wall thickness, in a similar subpopulation aged 17-25 years from South China, average thicknesses (width) of 0.73 mm, 0.66 mm, and 0.25 mm of the palatal canal wall and 1.03 mm, 0.79 mm, and 0.26 mm of the buccal canal wall, in coronal third, middle third, and apical third levels, respectively, were reported [7]. The discrepancies may be due to the differences in regions of China and diverse levels examined in the maxillary first premolars. Similar to the previous report [3], the width of the buccal wall was significantly larger than that of the palatal wall. As we know, posts must be surrounded by 1.0 mm of sound dentin to reinforce teeth. Based on the findings of anatomic features of the furcation grooves, to prevent vertical root fractures or perforations, it should be suggested to avoid posts in these roots or vigorously reducing the dentin width in endodontic or restorative procedures [25, 26].

The limitation of our study protocol was the somewhat small sample size in the examined furcation grooves using the micro-CT technique. Besides, the pulp cavity size and shape of maxillary first premolars showed significant differences in different age groups [27]. In our paper, furcation grooves of maxillary first premolars were measured in patients aged 15-25 years. It was suggested that the influence of age on the furcation groove reduced to some extent. However, further research is warranted to evaluate the anatomical morphology of the palatal furcation groove of the buccal root across different age groups with larger sample size in maxillary first premolars.

## 5. Conclusion

In conclusion, based on the CBCT method, the more common types were 1 root and 2 roots, in which the root canal type IV is mainly distributed in maxillary first premolars. Root bifurcations were found and distributed mostly in the middle third level in the general population. Notably, the palatal furcation groove indicated irregularity with different lengths, depths, and widths by the micro-CT technique in adolescent samples. These findings may help dentists to understand the anatomical morphology and to improve the outcomes of endodontic treatments.

## Figures and Tables

**Table 1:** Distribution of root types of maxillary first premolars according to sex and position (%).

	Patients (*n*)	Teeth (*n*)	1 root	2 roots	3 roots
Sex					
Male	212	424	260 (61.32)^a^	162 (38.21)^a^	2 (0.47)
Female	228	456	358 (78.51)^b^	96 (21.05)^b^	2 (0.47)
Position					
Left lateral teeth	440	440	306 (69.54)^NS^	132 (30.00)^NS^	2 (0.46)
Right lateral teeth	440	440	312 (70.90)^NS^	126 (28.64)^NS^	2 (0.46)
Total	880	880	618 (70.22)	258 (29.32)	4 (0.46)

^a,b^The comparison of roots between male and female patients with *P* < 0.05; ^NS^the comparison of roots between the left and right teeth with no statistical difference.

**Table 2:** Frequency of root canal types of maxillary first premolars (%).

Roots	Types of canal configuration^†^								
	I (%) (1-1)	II (%) (2-1)	III (%) (1-2-1)	IV (%) (2-2)	V (%) (1-2)	VI (%) (2-1-2)	VII (%) (1-2-1-2)	VIII (%) (3-3)	Total
1 root	245 (27.84)	181 (20.57)	9 (1.02)	134 (15.23)	29 (3.30)	13 (1.48)	9 (1.02)	—	620
2 roots	—	—	—	256 (29.09)	—	—	—	—	256
3 roots	—	—	—	—	—	—	—	4 (0.46)	4
Total	245 (27.84)	181 (20.57)	9 (1.02)	390 (44.32)	29 (3.30)	13 (1.48)	9 (1.02)	4 (0.46)	880

^†^Vertucci's classification of canal system.

**Table 3:** Measurement sites in the vertical and horizontal planes (mm).

Parameters	A1B1	C1D1	A1C1	B1D1	DE		
					Root coronal	Root middle	Root apical
Minimum	3.14	1.02	0	0	0.25	0.21	0.08
Maximum	9.30	7.63	0.62	4.65	1.59	0.86	0.35
Mean	5.78	3.51	0.05	2.23	0.57	0.47	0.22
SD	1.79	2.17	0.18	1.70	0.32	0.17	0.07

Note: A1B1 represents the length from the root DE represents furcation position to the apical position; C1D1 represents the length from the start point to the end point of the furcation groove; A1C1 represents the length from the root furcation position to the start point of the furcation groove; B1D1 represents the length from the apical position to the end point of the furcation groove; furcation groove depth of different levels.

**Table 4:** Thickness of root canal wall in different portions of buccal root (mm).

Parameters	Root coronal	Root middle	Root apical
Buccal aspect wall thickness (AB)	1.33 ± 0.21^a^	1.15 ± 0.21^a^	0.93 ± 0.31^a^
Palatal aspect wall thickness (CD)	0.88 ± 0.22^b^	0.83 ± 0.16^b^	0.65 ± 0.31^b^

^a,b^The comparison of thickness between buccal aspect and palatal aspect wall with *P* < 0.05.

## Data Availability

The CBCT and micro-CT images data used to support the findings of this study are restricted by the local institutional review board at the Air Force Medical University in order to protect patients' privacy.

## Additional Points

*Highlights*. (1) The most common canal configuration in maxillary first premolars was canal type IV (44.32%), followed by type I (27.84%) and type II (20.57%). (2) Root bifurcations had 40.13% prevalence which was distributed mostly in the middle third. (3) We found that 95.83% configuration had furcation grooves, with obvious changes for the length and mean depth in bifurcated maxillary first premolars.
